# Evaluation of Removal Force in Prosthetic Components of Morse Taper Dental Implants

**DOI:** 10.1590/0103-6440202205084

**Published:** 2022-10-21

**Authors:** Angelo Marcelo Tirado dos Santos, Glaykon Alex Vitti Stabile, Klissia Romero Felizardo, Sérgio Eduardo Ramos dos Santos, Sandrine Bittencourt Berger, Ricardo Danil Guiraldo, Alcides Gonini Gonini, Murilo Baena Lopes

**Affiliations:** 1 Department of Restorative Dentistry, University North of Parana, Londrina, PR, Brazil.; 2 Department of Oral Medicine and Pediatric Dentistry, State University of Londrina, Londrina, PR, Brazil.; 3 Department of Restorative Dentistry, State University of Londrina, Londrina, PR, Brazil.; 4 Department of Restorative Dentistry, Paranaense University, Londrina, PR, Brazil.

**Keywords:** dental implants, prosthesis failure, cone morse, tensile mechanics

## Abstract

The longevity of prosthetic rehabilitation is determined by the stability of the implant and abutment interfaces. True morse taper connections on dental restorations have been effective, however activation force still empirical. This work compared the activation strength and internal contact of Morse taper system according to the removal force. Eighty sets, composed of implants and prosthetic abutments, were evaluated with different internal contact areas; 15.12mm^2^ (G3.3) and 21.25mm^2^ (G4.3). The specimens were activated at 0° and 30°, with loads of 10, 20, 40 and 60N. The specimens were submitted to tensile test and the data to ANOVA and Tukey’s tests (α=0.05). Representative specimens were examined under SEM. Removal force of G3.3 (2.15±1.33MPa) did not differed to G4.3 (1.99±1.03MPa). The activation at 0º (2.95±0.98MPa) statistically differed to 30º (1.19±0.54MPa). The 60N load was statistically superior for G3.3 and there was no statistical difference between 20N to 60N in G4.3. The values of 10N at 30^o^ and 20N at the long axis of the morse taper implant, independent of the frictional contact area showed the best settlement.

## Resumo

A longevidade da reabilitação protética é determinada pela estabilidade das interfaces implante e pilar. Conexões de cone Morse em restaurações dentárias têm se mostrado eficazes, porém a força de ativação ainda é empírica. Este trabalho comparou a força de ativação e contato interno do sistema cone Morse de acordo com a força de remoção. Oitenta conjuntos, compostos por implantes e pilares protéticos, foram avaliados com diferentes áreas de contato interno; 15,12mm^2^ (G3.3) e 21,25mm^2^ (G4.3). Os corpos-de-prova foram ativados a 0° e 30°, com cargas de 10, 20, 40 e 60N. Os corpos de prova foram submetidos ao ensaio de tração e os dados aos testes ANOVA e Tukey (α=0,05). Espécimes representativos foram examinados em MEV. A força de deslocamento do G3.3 (2,15±1,33MPa) não diferiu do G4.3 (1,99±1,03MPa). A ativação a 0º (2,95±0,98MPa) diferiu estatisticamente para 30º (1,19±0,54MPa). A carga de 60N foi estatisticamente superior para G3.3 e não houve diferença estatística entre 20N a 60N no G4.3. Os valores de 10N em 30^o^ e 20N no longo eixo do implante cone morse, independente da área de contato friccional apresentaram o melhor assentamento.

## Introduction

The replacement of missing teeth with endosseous implants has become an integral part of modern dental health care. Different implant designs, implant-abutment connections, and prosthetic components, including different interfaces between implants and prosthetic connectors have been developed with the aim of achieving better performance with functional chewing loads ^1^. These many factors may interfere with prosthetic stability that can directly influence the development of future clinical protocols and can affect the osseointegration process ^2^. Without consensus, the connection options between endosseous implants and dental prosthesis are still the object of study and discussion in dental implantology.

The implant/abutment connection is generally described as an external or internal connection. The external hexagon connection was developed to facilitate implant insertion rather than to provide clinicians with an antirotational device ^3^. Therefore, the external hexagon might allow micromovements of the abutment under a high occlusal load, which may cause instability of the joint resulting in abutment screw loosening or fatigue fracture ^4^. Considering this, internal connections have been introduced to lower or eliminate these mechanical complications and reduce stress transferred to the crestal bone ^5^.

The biomechanics of dental implants with external and internal connection interfaces are different. Some of the mechanical failures reported in dental implantology are related to the screws loosening or prosthetic components and implants breaking ^6^ ^,^ ^7^ ^,^ ^8^ and it seems to be independent of the connection interface ^5^. Beside the osseointegration issues, the prosthetic screw is considered the weakest link in the whole prosthesis-implant set, principally in single-tooth prosthesis, since it receives high oblique loads during chewing ^9^. Furthermore, screw-retained prosthetic devices were considered a bad option that could cause inflammation and bone loss ^10^. Loosened abutment screws and prosthesis screws are often found at yearly clinical examinations ^10^. Thus, the long-term success of a prosthetic restoration supported by an osseointegrated implant is directly related to the precision of fit of the prosthetic components ^11^. This gives cause for concern during the rehabilitation ^12^.

The connection known as true Morse taper offer friction retention between their internal walls, discharging with the use of screws for joining the implant and prosthetic abutment ^13^. The resistance to abutment removal by axial and lateral forces is hypothetically derived from the principle that this connection is a fitting design in which a cone is embedded in another cone by friction, stablishing a solid tapered connection ^14^.

In this type of retention that depends on this friction fit, chewing compression force acts in the direction of abutment insertion, which favors interlocking union by self-activation of these conical interface implants ^15^ ^,^ ^16^. Some studies which evaluated single-tooth prosthetic rehabilitation using morse taper interface implants demonstrated great stability with only slight prosthetic removal percentages, varying from 0.37% to 1.7% ^13^ ^,^ ^17^.

The use of implant systems with this characteristic, associated or not to screws, presents some advantages such as: simplicity of the technique to make the prosthesis, the prosthetic component can be prepared; the neck profile of the prosthetic component is reduced in relation to the implant platform favoring a better esthetic; and decreased infiltration of microorganisms at the implant-abutment interface ^15^ ^,^ ^16^ ^,^ ^18^.

Considering that the activation of the prosthetic components on morse taper implants is performed by mallets, the torque variability in screws securing prosthetic components using manual tightening has been proven in several studies ^19^ ^,^ ^20^. At the anterior teeth, the activation along axis is possible, however is difficult to achieve that position for posterior teeth. There is necessity of apply the force to the long axis of the implant to activate the prosthetic components to avoid angular activations ^13^. The average maximum bite force, considering the influence of the position of the teeth along the dental arch and gender are 120 N for the central incisors; 117.52N for lateral incisors; 155 N for canines; 216.31N for the 1^st^ premolars; 248.68 for the 2^nd^ premolars; 270.26 N for the 1^st^ molars; and 258N for the 2^nd^ molars ^21^. Implants with frictional morse taper prosthetic interfaces are dependent on the correct activation but are driven by uncalibrated and unstandardized propulsion apparatus. These lead to the loss of retentive function of the implant-supported prostheses, a clinical condition that appears in practice daily.

This experimental study aimed to evaluate the tensile resistance (removal force) of morse taper system considering the activation force, verifying qualitatively the contact surfaces. The findings can give support to a future standardize of the activation force in these systems.

## Materials and methods

The study used 80 morse taper endosseous osseointegrated dental implants (Dental Implants Systems Kopp, Curitiba, PR, Brazil) and 80 solid straight trunnion prosthetic abutments (Dental Implants Systems Kopp, Curitiba, PR, Brazil) with 2.54º tapering within their walls. The dimensions of the implants and abutments are represented in Table 1. The sets were divided into two groups; 40 in the G3.3 group, with an internal contact area of 15.12mm^2^ and 40 in the G4.3 group with a contact area of 21.25mm^2^. The specimens were subdivided into 20 sets, which were activated at 0º and 30º; being 5 for each level of load activation studied (10, 20, 40 and 60N). To the sample size calculation was utilized the minimal difference between the means of treatments 5, the standard deviation 2, the number of treatments 5 and alpha 5% with a test power of 85%.

**Table 1:** Dimensions of the studied implants and prosthetics abutments.

	Group 3.3	Group 4.3
Implant External Diameter	3.3mm	4.3mm
Length of the Implant	11mm	11mm
Length of the Prosthetic Abutment	13mm	13mm
Prosthetic Connection Internal Diameter	2.0mm	3.0mm
Contact area	15.12mm^2^	21.25mm^2^

### Preparation and driving of the set samples

The implants were installed in standardized polyoxymethylene cylinders, (MGS - Engineering Plastics, Pinhais, PR, Brazil), of 15mm in diameter and 20mm in height, with a central perforation in the long axis (G3.3 - 2.8mm in diameter and 8mm deep and G4.3 - 3.8mm in diameter and 8mm deep). Using a hand wrench and a connector provided by the manufacturer, the implants were installed at 0º at depths of 8mm to simulate bone resorption of 3mm and coupled to metallic devices at 0º and 30º, according to ISO 1480.

**Figure 1:**
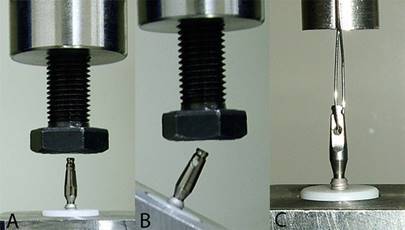
The specimen in the metallic device for activation at 0°(A) and at 30°(B). Application of tensile force (C).

To standardize and quantify the activation of the prosthetic component, the sets were activated in a universal testing machine (DL 2000 EMIC, São José dos Pinhais, PR, Brazil) at a speed of 30 mm/min, until reaching the loads predetermined for each subgroup (Figure 1A and 1B). This process was repeated three times sequentially in each of the activations, according to the recommendations the dental implant system manufacturer.

### Tensile test

After the activation of the specimens, the tensile force was measured in a universal testing machine at a speed of 1 mm/min at 0º (Figure 1C). The results were obtained in MPa and the retention force gain in percentage (Figure 1C).

### Scanning electron microscopy

The morphology of the retention area of the implant and prosthetic abutment were analyzed before and after the mechanical tests under a scanning electron microscope (20Kv, Magnification 60, Quanta 200, Philips) to evaluate possible deformation of the components.

### Statistical analysis

The means and standard deviation were calculated for each group. The means were submitted to Analysis of Variance of three factors: implant (frictional contact area), angulation and activation force, and the means differences analyzed by Tukey’s test (α=0.05).

## Results

There were no statistical differences in removal force between G3.3 (2.15±1.33MPa) and G4.3 (1.99±1.03MPa) groups. However, when the tensile test was evaluated, the activation at 0º (2.95±0.98MPa) was significantly larger than at 30º (1.19±0.54).

Greater frictional retention was observed with increasing activation force in both groups at 0º. Less resistance to removal was found when activated off the long axis of the implant. Higher mean values of prosthetic abutment retention was observed at 0º and 60N for both the G3.3 (4.72 MPa) and G4.3 (3.52 MPa) groups. Lower mean values were found for implants activated at 30º; 40N in the G3.3 group (0.92 MPa) and 30º; 20N in the G4.3 group (0.68 MPa). A retaining force gain toward the load activation (cold weld) was observed in all the sets activated at 0° and lower frictional retention in groups activated at 30º; 20, 40 and 60 N (Table 2).

In actuations of 0^o^ in the G3.3 group, the load of 60N was statistically superior to the others in the G3.3 group and there was no statistical difference between the activation loads of 10, 20, 40 N. The percentage of retention gain when the prosthetic component was activated was 273.70% for 10N and 89.50% for at 20N (Table 2). In actuations of 0^o^ in the G4.3 group, the removal force did not present statistical differences between the sets activated at 20, 40 and 60N. The higher gain of retention was found 306% for 10N and 174.50% for 20N (Table 2).

In actuations at 30º there were no statistical differences between load activations, for either, G3.3 or G4.3. However, it was observed that as higher the activation force as higher the loss of retention (Table 2).

**Table 2:** Means and standard deviation (SD) of resistance to the component removal in MPa and percentage of retention gain with different loads in comparison to the applied force to activate the components and activation angles.

		10N			20N		40N		60N	
		MPa	%	MPa	%	MPa	%	MPa	%
G3.3	0^o^	2.47±0.72 a	273,70	2.50±0.65 a	89,50	2.80±1.10 a	6,15	4.72±0.31 b	19,20
	30^o^	1.10±0.36 e	67,40	1.04±0.18 e	-21,10	0.92±0.41 e	-65,10	1.65±0.74 e	-58,23
G4.3	0^o^	1.91±0.28 c	306,00	2.58±0.43 cd	174,50	3.14±0.48 d	67,15	3.52±0.39 d	24,88
	30^o^	0.95±0.18 f	102,90	0.68±0.30 f	-27,50	1.36±0.32 f	-27,30	1.78±0.68 f	-36,65

Different letters indicate statistical difference by Tukey’s test at 5% of significant level

### Scanning electron microscopy

**Figure 2:**
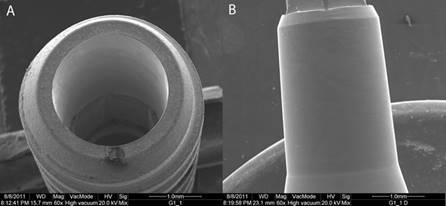
Implant’s scanning electron photomicroscopy (A) and prosthetic abutment (B) after mechanical tests at 0°. Small contact marks were observed on the prosthetic abutment extension.

**Figure 3:**
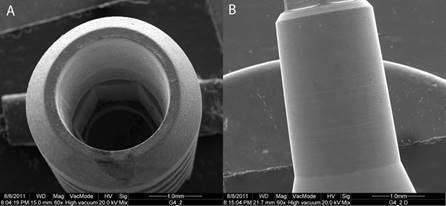
Implant’s scanning electron photomicroscopy (A) and prosthetic abutment (B) after mechanical tests at 30°. Small contact marks were observed on the prosthetic abutment apical region.

The analysis revealed discrete contact marks in the direction of activation in the prosthetic abutment, located throughout the area when activated at 0º (Figure 2) and more in the apical region when activated at 30º (Figure 3). There was no evidence of any significant deformation, cracks or fractures in the structure of the implant or the prosthetic abutment.

## Discussion

Mechanical failures reported in dental implantology vary from screws loosening to breaking of the prosthetic components and implants. The analysis of stress concentrations around implants, abutments and peri-implantal bone structures is extremely important for a good clinical prognosis of dental implant rehabilitation ^22^. Some clinical studies have verified unfastening of the prosthetic screws of between 30.7% and 49% of external interface implants type (7, 8). The prosthetic screws are considered the weakest link in the whole implant-prosthesis set because of the high oblique loads during chewing they receive, which make them a concern during rehabilitations ^12^.

In prosthetic connections using screws, their preloading is a determining factor for retention because the implant and prosthetic abutment relationship occurs through the overlapping connections ^23^. Screw cone and morse taper connections, considered as highly stable or self-locking, have great capacity to bear occlusal loads because the installation of the prosthetic abutment to the implant promotes a close fit between their surfaces ^15^ ^,^ ^18^. Conversely, the loss of the preload force in screw cone implants proved to be similar to external hexagonal interface implant system after cycling tests ^6^. Another study ^12^ comparing implants with external hexagonal, internal hexagonal and conical connections, found worse results for the external connections.

Several clinical researches ^13^ ^,^ ^17^, which evaluated single-tooth prosthetic rehabilitations using morse taper interface implants, confirmed greater stability and less prosthetic removal displacement. The retention through friction in true morse taper interface implant/abutment is achieved when no space exists between the mating components and the parts are forced together ^24^. An interlock occurs between the prosthetic abutment and implant surfaces which gives rise to mechanical union of the set which in turn causes dental prosthesis retention and stability ^16^. In the present study, a proportional increase in the activation load provided an increase in retention of the prosthetic component to the implant. This was verified for all specimens activated to the long axis of the implant.

The required force to displace the prosthetic component in true morse taper dental implants system was higher when applied to the long axis of the implant, compared to 30º activation. On the other hand, higher activation force at 0º in both groups showed better interlock of the prosthetic abutment to the implant implying in higher resistance to set removal. These results demonstrate the importance to correct the position of the intrabuccal activation device (beat connection) in these implants. To the manufacturing of an intrabuccal “jig” device to guide the activations of prosthetic components or dental crowns to the implant’s long axis could avoid incorrect activations and future inadvertent removal displacement of the prosthetic dental element ^13^.

The group II with the larger contact area did not present statistical difference when compared to the group I with the smaller area contradicting Norton ^16^, where screw cone interface implants and larger surfaces promoted greater resistance to removal displacement in single-tooth dental prosthesis. In this way, lower diameter implants could be preferable. Besides, these lower diameter implants promote an area of better irrigation around themselves, promoting better osseo-integration ^25^. The choice for different morse taper implant diameters did not bring differences at the prosthetic emergence profile, as the components for different diameters are the same.

The sets of implant activation between 10 and 60N on the long axis showed no statistic difference among all loads except by 60N, which showed the greatest prosthetic abutment retention in G3.3. When the G4.3 were evaluated, all groups showed no statistical differences between them, except by the 10N group, which showed lower results. However, the activation, which promoted better retention in percentage, was 10 and 20N load, with variation respectively of 273.70% and 89,50% for G3.3 and 306% and 174,5% for G4.3. Within the limits of work and clinically extrapolating, during fixation of prostheses that settle to the long axis of the tooth, the 20N load would be the most appropriate, because it works independent of the implant diameter. For difficult access regions, where activation at the long axis is impossible, the load of 10N can be more adequate, as it also provides appropriate component activation, probably due to the transversal power dissipation. The physiologic masticatory forces involved in the chewing process generates forces that may vary from 178.54 to 294.3N ^15^ ^,^ ^16^ ^,^ ^21^, which after initial stabilization, could promoting self-activation of the entire prosthetic system ^18^.

The analysis using scanning electron microscope at 0^o^ verified small contact marks in the retention cone’s surface occurring because of the friction of the prosthetic abutment (Figure 2). No deformations were detected in the friction region or neighboring areas. In the groups activated at 30º, marks were located more apically to the retention cone, which evidenced possible contact loss of the interlock; this is probably due to transversal forces (Figure 3).

Despite the results of the present study suggest the reference values of 10N and 20N for activation at 30^o^ and the longa axis, the currently available devices do not allow quantification of the frictional system activation force at clinical environment as torquimeters used in screw dependent dental implant systems. Since some studies ^19^ ^,^ ^20^ have shown that the use of manual keys without a measuring instrument for dental implant screwed systems presented variability, this study evidencing that the morse taper interface implant system also suffers from discrepancies between drive load and in this way, providing some parameters to define the activation load in morse taper prosthetic implant abutments and the implications of angled drive to obtain better clinical prognosis.

## Conclusions

The values of 10N at 30^o^ and 20N at the long axis of the morse taper implant, independent of the frictional contact area, considering the oral conditions showed the best settlement.
